# Synthesis, spectroscopic, coordination and biological activities of some organometallic complexes derived from thio-Schiff base ligands

**DOI:** 10.1016/j.saa.2013.06.078

**Published:** 2014-01-03

**Authors:** Azza A. Abou-Hussein, Wolfgang Linert

**Affiliations:** a Faculty of Women for Arts, Science and Education, Ain Shams University, Heliopolis, Cairo, Egypt; b Institute of Applied Synthetic Chemistry, Vienna University of Technology, Getreidemarkt 9/163-AC, 1060 Vienna, Austria

**Keywords:** Monoacetylferrocene, 1,1′-Diacetylferrocene, Thio-Schiff base, Metal complexes, Spectroscopy, Biological activity

## Abstract

Two series of mono- and binuclear complexes cyclic or acyclic thio-ferocine Schiff base ligands, derived from the condensation of 2-aminobenzenthiol (L) with monoacetyl ferrocene in the molar ratio 1:1 or in the molar ratio 1:2 for diacetyl ferocine have been prepared. The condensation reactions yield the corresponding Schiff Base ligands, HLa-Maf and H_2_Lb-Daf. The chelation of the ligands to metal ions occurs through the sulfur of the thiol group as well as the nitrogen atoms of the azomethine group of the ligands. HLa-Maf acts as monobasic bidentate or dibasic tetradentate, while H_2_Lb-Daf behaves as twice negatively cargend tetradentate ligand. The structures of these ligands were elucidated by elemental analysis, infrared, ultraviolet–visible spectra, as well as ^1^H NMR spectra. Reactions of the Schiff bases ligands with ruthenium(III), oxovanadium(IV) and dioxouranium(VI) afforded the corresponding transition metal complexes. The properties of the newly prepared complexes were analyse by elemental analyses, infrared, electronic spectra, ^1^H NMR as well as the magnetic susceptibility and conductivity measurement. The metal complexes exhibits different geometrical arrangements such as octahedral and square pyramidal coordination. Schiff base ligands and their metal complexes were tested against two pathogenic bacteria as Gram-positive and Gram-negative bacteria as well as one kind of fungi to study their biological activity. All the complexes exhibit antibacterial and antifungal activities against these organisms.

## Highlights

• Thio Schiff base complexes derived from mono- and diacetyl ferrocene were synthesized.
• The complexes are characterized by different spectroscopic techniques.
• The complexes have different varieties of geometrical structures.
• Biochemical studies were studied.

## Graphical abstract

**Figure:**
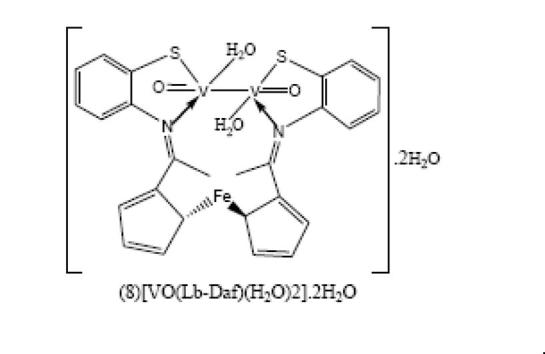

## Introduction

π-Conjugated organic molecular systems provide a wide range of possible substitution and functionality are of considerable interest because of their applications in biochemistry [1], [2] asymmetric catalysis [3], organometalic chemistry [4], [5], [6], [7], [8], [9], [10] material science [11], [12], [13], electronic and photonic technologies relevant to optical computing applications [14]. The redox-active ferrocene moiety has also been exploited in the electrochemical sensing of anions; these receptors are expected to show cathodic shifts in their redox process when complexed to an anion [15], [16], [17]. Furthermore, It has been reported, [18], [19] the use of platinum and gold complexes of 1,1′-bis(diphenylphosphino)ferrocene might be used successfully against various tumours. An enhanced antibiotic activity of penicillin and cephalosporine may be obtained by replacing the aromatic group with the ferrocenyl moiety [20], [21].

In view of these facts, we have previously synthesized a new class of mono and disubstitutedferrocene-derived from thio-Schiff bases ligands and their coordination behaviour with different transition metals for which antimicrobial activities have been discussed [22]. In continuation of our work, two new series of cyclic or acyclic mono- or binuclear complexes have been prepared from thio-Schiff base ligands, HLa-Maf and H_2_Lb-Daf. The reaction of Schiff bases ligands with ruthenium(III), oxovanadium(IV) and dioxouranium(VI), affords a mono or binuclear complex of a cyclic or macrocyclic complexes, according to the mole ratio of metal to ligand. The Schiff base and their newly prepared metal complexes were identified by different physicochemical and spectroscopic techniques. The microbial activity of Schiff bases and their transition metal complexes have been investigated against two pathogenic bacteria (*Staphylococcus aureus*) as Gram-positive bacteria, and (*Pseudomonas fluorescens*) as Gram-negative bacteria in addition to one kind of fungi (*Fusarium oxysporum*) to assess their antimicrobial properties.

## Experimental

### Materials

The nitrate salt of dioxouranium(VI) was obtained from Merck or DBH. Oxovanadium(IV) acetae monohydrate is BDH. Ruthenium(III) chloride trihydrates was purchased from Sigma. Organic solvents (absolute ethyl alcohol, methyl alcohol, acetone, dimethylformamide (DMF) and dimethylsulfoxide, (DMSO) are reagent grade and were used without further purification. Mono- and 1,1′-diacetylferrocene were punctured from Aldrich.

### Physical measurements

Microanalyses of carbon, hydrogen and nitrogen were carried out on a Perkin–Elmer 2400 Series II Analyzer. Electronic spectra of DMF solutions of the metal complexes were carried out by using UV–Vis Perkin–Elmer Model Lamda 900. NIR IR and Mid-range. FTIR spectra of the compounds were recorded as KBr-pellets within the range 4000–400 cm^−1^ using a Perkin–Elmer 16PC FTIR spectrometer. Far FTIR spectra were recorded within the range 600–200 cm^−1^ on a Perkin–Elmer System 2000, spectrometer using polyethylene pellets at Institute of Applied Synthetic Chemistry, Vienna University of Technology. Analyses of the metals in the complexes were carried out by the dissolution of the solid complex in concentrated HNO_3_, neutralizing the diluted aqueous solutions with ammonia. The metal content of the solutions were then titrated with EDTA [23]. 0.1 g of uranyl complex was placed in a clean and dry weighed crucible and ignited on Bunsen flame for 15 min. After that, the crucible was ignited in a muffle oven and heated at 1000 °C until constant weight for about 2 h. The residue was cooled and weighed again as U_3_O_8_. Mass spectra measurements were carried out on a Shimadzu-GC–MS-QP, mass spectrometer model 1000 EX using a direct inlet system, at 220 °C and 70 eV in the Micro Analytical Center, Cairo University, Egypt. ^1^H NMR spectra of the ligands and UO_2_(IV) complexes, as solutions in DMSO-*d*_6_, were recorded on a Bruker WP 200 SY, spectrometer at room temperature using TMS as an internal standard, at national research center, Giza, Egypt. Magnetic susceptibilities of the complexes were measured at room temperature using a Johnson Matthey, Alfa Products, model MKI magnetic susceptibility balance. The effective magnetic moments were calculated from the expression *μ*_eff_. = 2.828 (χ_M_·T)^1/2^ B.M., where χ_M_ is the molar susceptibility corrected using Pascal’s constants for the diamagnetism of all atoms in the compounds [24]. Molar conductivities were measured in DMF solutions of the complexes (10^−3^ M) using a model LBR, WTWD-812, Weilheim Conductivity meter fitted with a LTA100 cell.

### Synthesis of the Schiff base, HLa-Maf and H_2_Lb-Daf

The Schiff base, HLa-Maf and H_2_Lb-Daf ligands were prepared by the addition of monoacetyl ferrocine, Maf, (1.00 g, 4.38 mmol) in ethanol (20 mL) to 2-aminobenzenthiol (0.548 g, 4.38 mmol) in a molar ratio 1:1, (For details see Supplementary material) The species H_2_Lb-Daf was synthesized in the molar ratio, 1:2 by the addition of 1,1′-diacetyl ferrocine, Daf, (1.00 g, 3.70 mmol) in ethanol (20 mL) to (1.00 g, 3.70 mmol) (2-aminobenzenthiol). The solutions were refluxed for 3 h. Brown (HLa-Maf) or dark orange (H_2_Lb-Daf) crystals were formed on cooling the solutions slowly to room temperature. The precipitates were collected by filtration, washed with ethanol then diethylether and finally air-dried. The yields were 68.97%, m.p. 246 °C for HLa-Maf and 62.46%, m.p. 234 °C for H_2_Lb-Daf.

### Synthesis of the transition metal complexes of the Schiff base

Reaction of the Schiff base ligand HLa-Maf with ruthenium(III), oxovanadium(IV) and dioxouranium(VI) ions in molar 1:1 and 2:1 afforded the corresponding stoichiometry transition metal complexes. On the other hand, mono and binuclear complexes for H_2_Lb-Daf were obtained in its binegative form in the molar ratio 1:1 and 1:2 (H_2_Lb-Daf: metal). One example for the detailed preparations is given for each method to obtain mono- or binuclear metal complex in acyclic or cyclic geometry. For the preparation of the VO^2+^, complex, 0.1 g of sodium acetate was added as a buffering agent to raise the pH medium.

#### Synthesis of transition metal complexes **1** and **4**

A solution of Ru(III) (2.00 g, 9.64 mmol) in ethanol (40 mL) was added gradually to a solution of the corresponding amount of Schiff base, HLa-Maf (3.23 g, 9.46 mmol), i.e. in the molar ratio, 1 metal: 1 HLa-Maf and to (6.46 g, 6.20 mmol) for (HLa-Maf)_2_ in ethanol (20 mL), i.e. in the molar ratio, 1 metal: 2(HLa-Maf). The solutions were stirred for 30 min and heated under reflux for further 3 h. The precipitate was formed after cooling to room temperature. The precipitate was filtered off, washed with ethanol, and then diethyl ether and finally air dried. The yields were 58.76%, m.p. > 250 °C and 65.43%, m.p. > 250 °C for, **1**[Ru(La-Maf)(Cl)_2_(H_2_O)_2_]·H_2_O and (4) [Ru(La-Maf)_2_(H_2_O)_2_]·H_2_O·Cl complexes, respectively (For details see Supplementary material).

#### Synthesis of the transition metal complexes **7** and **11**

A solution of Ru(III) (1.00 g, 4.82 mmol) in ethanol (40 mL) was added gradually to solution of the corresponding amount of Schiff base, H_2_Lb-Daf (2.33 g, 4.82 mmol), i.e. in the molar ratio, 1 metal: 1 H_2_Lb-Daf and to (1.167 g, 2.41 mmol) for (H_2_Lb-Daf)_2_ in ethanol (20 mL), i.e. in the molar ratio, 2 metal: 1 H_2_Lb-Daf. The solutions were stirred for 30 min and heated to reflux for 3 h. The precipitate was formed after cooling to room temperature. It was filtered off, washed with ethanol, and then diethylether and finally air dried. The yields were 54.82%, m.p. > 250 °C and 58.46%, m.p. > 250 °C for (7)[Ru(Lb-Daf)(Cl)(H_2_O)]H_2_O and (10) [Ru_2_(Lb-Daf)(Cl)_4_(H_2_O)_2_]·H_2_O complexes, respectively (For details see Supplementary material).

### Biological studies

In vitro antibacterial activity studies were carried out using the standardized disc-agar diffusion method [25] to investigate the inhibitory effect of the synthesized ligand and complexes against Gram-positive bacteria, such as *S. aureus* (ATCC25923), Gram-negative bacteria: as *P. fluorescens* (S97) and *F. oxysporum* as a kind of fungi. The antibiotic *chloramphencol* was used as standard reference in the case of Gram-negative bacteria and *cephalothin* was used as standard reference in the case of Gram-positive bacteria and *cycloheximide* was used as standard antifungal reference. An inhibition zone diameter indicates that the tested compounds are active against the used kinds of the bacteria and fungus. The tested compounds were dissolved in DMF (which have no inhibition activity), to get concentrations of 2 and 1 mg mL^−1^. The test was performed on medium potato dextrose agar (PDA) which contains infusion of 200 g potatoes, 6 g dextrose and 15 g agar [26], [27]. Uniform size filter paper disks (3 disks per compound) were impregnated by equal volume (10 mL) from the specific concentration of dissolved tested compounds and carefully placed on incubated agar surface. After incubation for 36 h at 27 °C in the case of bacteria and for 48 h at 24 °C in the case of fungi, inhibition of the organisms, which evidenced by clear zone surround each disk, was measured and used to calculate mean of inhibition zones. The activity of tested compounds was categorized as (i) low activity = mean of zone diameter is ⩽1/3 of mean zone diameter of control, (ii) intermediate activity = mean of zone diameter ⩽2/3 of mean zone diameter of control and (iii) high activity = mean of zone diameter >2/3 of mean zone diameter of control. The studies are carried out at Faculty of Agriculture, Department of plant Pathology, Al-Azhar University.

## Results and discussion

The physical and analytical data of the Schiff base ligands and their corresponding transition metal complexes are listed in Table 1. Comparing the IR spectra of the complexes with the spectra of the free ligands elucidated the mode of binding of the Schiff bases to the metal ions. These complexes are investigated also by elemental analyses and different spectroscopic methods.

**Table 1:** Physicochemical properties of the Schiff base HLa(Maf), H_2_Lb(Daf) ligands and their transition metal complexes.

Ligand/complex	M.F.	M.Wt.	Yield (%)	Color	D.P. °C	Elemental analysis, Calc. (found)			
						%C	%H	%N	%M
**I HLa(Maf)**	C_18_H_17_FeNS	335.043	68.97	Orange	173	64.46	5.11	4.17	–
						(64.21)	(5.41)	(3.94)	
(1)[Ru(La-Maf)(Cl)_2_(H_2_O)_2_]·H_2_O	C_18_H_22_Cl_2_FeNO_3_RuS	559.909	58.76	Black	>250	38.57	3.95	2.50	18.20
						(38.28)	(3.47)	(2.75)	(18.93)
(2)[VO(La-Maf)(OAc)(H_2_O)]·H_2_O	C_20_H_23_FeNO_5_SV	496.008	58.48	Green	>250	48.38	4.67	2.82	10.27
						(48.73)	(4.16)	(2.53)	(9.96)
(3)[UO_2_(La-Maf)(H_2_O)_2_]·NO_3_	C_18_H_20_FeN_2_O_7_SU	702.084	67.56	Orange	>250	30.76	2.87	3.98	33.90
						(30.47)	(2.59)	(3.47)	(34.43)
(4)[Ru(La-Maf)_2_(H_2_O)_2_]·H_2_O·Cl	C_36_H_38_ClFe_2_N_2_O_3_RuS_2_	858.975	65.43	Black	>250	50.29	4.45	3.26	11.86
						(50.74)	(4.86)	(3.82)	(12.31)
(5)[VO(La-Maf)_2_H_2_O]·H_2_O	C_36_H_36_Fe_2_N_2_O_3_S_2_V	771.030	64.83	Green	>250	56.02	4.70	3.63	6.60
						(56.49)	(4.56)	(3.27)	(6.92)
(6)[UO_2_(La-Maf)_2_]·3H_2_O	C_36_H_38_Fe_2_ N_2_O_5_S_2_U	992.142	72.43	Orange	>250	43.54	3.86	2.82	23.99
						(43.16)	(3.37)	(2.64)	(23.86)
**II H_2_Lb**(**Daf**)	C_26_H_24_FeN_2_S_2_	484.46	62.46	Broun	187	64.46	4.99	5.78	–
						(64.83)	(4.56)	(5.37)	
(7)[Ru(Lb-Daf)(Cl)(H_2_O)]·H_2_O	C_26_H_26_ClFeN_2_O_2_RuS_2_	654.951	54.82	Black	>250	47.63	4.00	4.27	15.55
						(47.18)	(4.45)	(4.69)	(14.35)
(8)[VO(Lb-Daf)]·2H_2_O	C_26_H_26_FeN_2_O_3_S_2_V	585.01	66.21	Black	>250	53.33	4.47	4.78	8.70
						(53.77)	(4.73)	(4.28)	(8.25)
(9)[UO_2_(Lb-Daf)]·2H_2_O	C_26_H_26_FeN_2_O_4_S_2_U	788.119	57.11	Orange	>250	39.58	3.32	3.55	30.20
						(39.14)	(3.34)	(3.78)	(30.58)
(10)[Ru_2_(Lb-Daf)(Cl)_4_(H_2_O)_2_]·H_2_O	C_26_H_28_Cl_2_FeN_2_O_3_Ru_2_S_2_	809.835	67.57	Black	>250	38.52	3.48	3.45	25.16
						(38.73)	(3.74)	(3.63)	(26.28)
(11)[(VO)_2_(Lb-Daf)(H_2_O)_2_]·2H_2_O	C_26_H_30_FeN_2_O_6_S_2_V_2_	687.977	58.46	Dark green	>250	45.35	4.39	4.07	14.80
						(45.26)	(4.64)	(4.46)	(14.47)
(12)[(UO_2_)_2_(Lb-Daf) (H_2_O)_4_]·2NO_3_	C_26_H_30_FeN_4_O_14_S_2_U_2_	1218.15	56.68	Orange	>250	25.61	2.48	4.59	39.08
						(25.37)	(2.63)	(4.16)	(38.27)

### Characterization of Schiff bases ligands, HLa-Maf and H_2_Lb-Daf

The Schiff base ligands, HLa-Maf and H_2_Lb-Daf, were characterized by elemental analysis, ^1^H NMR spectroscopy and UV–Vis spectrophotometer in DMF and in the solid state by FT-IR. The vibrational frequencies and tentative assignments for free HLa-Maf and H_2_Lb-Daf ligands and their transition metal complexes were recorded and are given in Table 2. Three important findings in the infrared spectrum of ligands will be discussed. The first one is the disappearance of the stretching frequencies of *υ*(NH_2_) bands which observes at 3285 and 3267 cm^−1^ in 2-aminobenzenthiol as well as stretching frequencies of carbonyl groups, *υ*(C=O) (ca. 1723 cm^−1^) of mono or diacetyl ferocine as a result of the condensation reactions [28], [29]. The second feature is the appearance of two intense band at 1655 cm^−1^ and 1573 cm^−1^ for HLa-Maf and at 1663 and 1567 cm^−1^ for H_2_Lb-Daf, corresponding to the azomethine groups, *υ*(C=N) and *υ*(C=C), stretching frequencies respectively. The medium broad band at the region 2586 cm^−1^ can be assigned to —SH group. The broadening may be due to the intermolecular hydrogen bonded N⋯HS [30], [31]. The characteristic frequencies of the ferrocenyl moiety in the spectra of the two Schiff base ligands were observed near 3068, 1410 and 1156 cm^−1^ and are assigned to a C—H stretching band, asymmetric C—C vibration and asymmetric ring-breathing vibration, respectively. The two bands located at 1014 and 823 cm^−1^ are assigned to parallel and perpendicular C—H bands, respectively. The remaining characteristic band at 484 cm^−1^ can be assigned to the Fe–Cp stretching frequency [32], [33]. ^1^H NMR spectra of HLa-Maf and H_2_Lb-Daf confirm the absence of NH_2_ group (ca. 4.92) as a result of the condensation of the amine with the carbonyl groups. The signals due to methyl groups (—CH_3_), thiol group (—SH) and phenyl ring are observed at 2.23, 3.52 and (6.47–7.68) for HLa-Maf and at 2.18 and 4.48 and (6.48–7.77) for H_2_Lb-Daf, respectively [34]. The spectra of HLa-Maf, Schiff base ligand, display signals due to α and β the protons of the cyclopentadienyl rings at 4.47 and 4.41 assigned to [m, 4H, C_5_H_4_] and resonance at 4.06 for [s, 5H, C_5_H_5_-Cp]. On the other hand, cyclopentadienyl rings of H_2_Lb-Daf, displayed multiplets signals at 4.82, 4.78, 4.61 and 4.45 due to α, α′, β and β′-protons of the cyclopentadienyl rings. [35], [36].

**Table 2:** Infrared frequencies of the main characteristic bands of the Schiff base ligand HLa-(Maf), H_2_Lb-(Daf) and transition metal complexes.

Ligand/complex		*ν*(H_2_O)	*ν*(C=N)	*ν*(SH)	*ν*(C—N) and	*ν*(C—S)	*ν*(M—N)	*ν*(M—S)	Other assignments
**I**	HLa(Maf)	–	1655s	2586 m	1263 m	764 m	–	–	–
(**1**)	[Ru(La-Maf)(Cl)_2_(H_2_O)_2_]·H_2_O	3350 br	1642 s	–	1243 m	751 m	435 m	374 m	326 *ν*(Ru—Cl)
(**2**)	[VO(La-Maf)(OAc) H_2_O]·H_2_O	3445 s, br	1645 s	–	1246 m	756 m	446 m	364 m	1575 *ν*as, 1355 *ν*sym
									Unidentate (Ac^−^) group
(**3**)	[UO_2_(La-Maf)(H_2_O)_2_]·NO_3_	3378 s	1634 s	–	1254 m	752 m	440 m	381 m	1383s, 856 m ionic NO_3,_
(**4**)	[Ru(La-Maf)_2_(H_2_O)_2_]·H_2_O·Cl	3500 s, br	1647 s	–	1258 m	758s	463 m	366 m	Ionic complex
(**5**)	[VO(La-Maf)_2_H_2_O]·H_2_O	3426 s, br	1639 s	–	1249 m	760s	464 m	364 m	970s, 958 m *ν* (VO)
(**6**)	[UO_2_(La-Maf)_2_]·3H_2_O	3356 s, br	1640 s	–	1246 m	756s	446 m	366 m	910 m, 897 *ν*_as_(UO_2_) & *ν*_s_(UO_2_),
**II**	H_2_Lb(Daf)	–	1663s	2586 m	1257 m	746s	–	–	–
(**7**)	[Ru(Lb-Daf)(Cl)(H_2_O)]H_2_O	3446 s	1658 s	–	1143 m	792 m	467 m	367 m	346, *ν*(Ru—Cl)
(**8**)	[VO(Lb-Daf)]·2H_2_O	3503 s,br	1655 s	–	1140 m	816 m	453 m	363 m	976s, 985 m *ν* (VO)
(**9**)	[UO_2_(Lb-Daf)]·2H_2_O	3458 s, br	1658 s	–	1142 m	826 m	460 m	386 m	915 m, 890 *ν*_as_(UO_2_) & *ν*_s_(UO_2_)
(**10**)	[Ru_2_(Lb-Daf)(Cl)_4_(H_2_O)_2_]·H_2_O	3452 s, br	1656 s	–	1148 m	835 m	458 m	–	335 *ν*(Ru—Cl)
(**11**)	[(VO)_2_(Lb-Daf)(H_2_O)_2_]·2H_2_O	3428s,br	1644 s	–	1147 m	786 m	456 m	376 m	973s, 989 m *ν* (VO)
(**12**)	[(UO_2_)_2_(Lb-Daf) (H_2_O)_4_]·2NO_3_	3464s	1641m	–	1150 m	791 m	465 m	362 m	1383 s, 856 m ionic NO_3_ group

s = strong, w = weak, m = medium, and br = broad,

### Characterization of the complexes of the Schiff base ligand

#### Infrared spectra

Infrared spectra of the complexes were recorded to confirm their structures. The vibration frequencies and their tentative assignments for HLa-Maf and H_2_Lb-Daf ligands and their transition metal complexes are listed in Table 2. The assignments were aided by comparison with the vibrational frequencies of the free ligand and their related compounds. There are four main features in the infrared spectra of the complexes. The first one is the shift of the stretching frequencies of the azomethine (—C=N—) group of the transition metal complexes to lower frequencies in the range, 1640–1655 cm^−1^, compared with free HLa-Maf and H_2_Lb-Daf, due to the coordination of the azomethine moiety, *ν*(C=N) vibration [37]. Further evidence of the bonding is given by the observation of new bands in the spectra of the metal complexes of medium or weak intensity at the region 467–435 cm^−1^ due to *ν*(M—N) stretching vibrations supporting the involvement of the nitrogen atom of the azomethine group via coordination [38], [39]. The second feature is the disappearance of the band assigned to the stretching vibration of —SH group upon complexation. This indicates the deprotonation of the thiol groups due to coordination with the metal ions [40]. This is further supported by the band around 752–746 cm^−1^ in the metal complexes due to *ν*(C—S) and is inconsistent with the appearance of new weakly to medium bands in the region 356–382 cm^−1^, which could be assigned to the stretching frequencies of *ν*(M—S) bands, respectively, confirmed that, the chelation to the metal ions is achieved thiol-sulphur atoms [41]. The third feature of the spectra of some complexes is the appearance of a broad band between the ranges 3300–3500 cm^−1^, which could be assigned to the stretching frequencies of the hydroxyl group of either crystalline or coordinated water molecules associated with the complex [42]. This result is confirmed by the elemental analysis. The later feature in the coordination behavior is associated with the anions, nitrate, chloride and acetate, respectively. For acyclic and macrocyclic ruthenium complexes, 1, **4**, **7** and **10**. The coordination behavior of chloride ions were investigated by the addition of AgNO_3_ solution for Ru(III) complexes, the chloride ions are detected only in complex **4**, [Ru(La-Maf)_2_(H_2_O)_2_]·H_2_O·Cl, where its ions were precipitated. It is worth mentioning that, the absorptions at 326, 346 and 335 cm^−1^ in the spectra of Ru(III) complexes **1**, **7** and **10** were attributed to *ν*(Ru—Cl) vibrations [43]. The oxovanadium complexes**, 2, 5** and **8** exhibit a strong band around 978–985 cm^−1^ .This reflects the high π-band order of vanadium to oxygen link of VO^+2^ and indicate the presence of monomeric oxovanadium species [44], [45]. On the other hand, for complex **11**, a high intensity band at 990 cm^−1^ is observed. This could be attributed to dimerisation via *V* = 0 which would be reflected by shifting the respective vibration [46], [47]. Bands in the region of 1575 cm^−1^ and 1335 cm^−1^ region for [VO(La-Maf)(OAc)(H_2_O)]·H_2_O, **2,** are assigned to *v*_as_(COO) and *v*_sym_(COO)] respectively. The difference between the frequencies of the two bands is suggestive of mono-dentate behavior of acetate moiety [48]. Also, the IR spectra of all the UO_2_(VI) complexes display a strong band around the ranges, 950–910 and 890–897 cm^−1^ which are assigned to the *ν*_as_(UO_2_) and *ν*_s_(UO_2_) modes, respectively. This observation suggests that the O=U=O moiety is virtually linear in these complexes [49], [50]. Additionally it has been observed that, dioxouranium(VI) complexes **3** and **12**, exhibited two bands at 1384 and 852 cm^−1^, respectively, due to the free nitrate [51], [52].

Concluding from the infrared data, HLa-Maf acts as monobasic bidentate ligand and coordinates to metal ions via the azomethine and thiol atoms. H_2_Lb-Daf, act as tetradentate binegative ligand through similar coordination centers. The corresponding frequencies of the ferrocenyl moiety of the complexes appeared at nearly the same position, which indicates that the cyclopentadienyl ring of the ferrocene is not directly coordinated to the metal ion.

### Electronic spectra, magnetic moments and molar conductivity measurement

The electronic spectra of the ligands and their transition metal complexes in the solid state with their assignments, magnetic moment and molar conductivity measurement are given in Table 3.

**Table 3:** Electronic absorption bands (nm), magnetic moments (B.M.) and molar conductivities (Ω^−1^ cm^2^ mol^−1^) transition metal complexes.

Complex		Electronic absorption bands their assignment, magnetic moments and molar conductivities			
		**d–d**^a^ Transition	**d–d** Transition assignment	*μ_complex_* (B.M.)	(*Λ*^b^)
(**1**)	[Ru(La-Maf)(Cl)_2_(H_2_O)_2_]·H_2_O	643(0.22)	^2^T_2g_ → ^2^A_2g_(*ν*_1_)	1.77	36
(**2**)	[VO(La-Maf)(OAc)(H_2_O)]·H_2_O	724(0.38)	^2^B_1g_ → ^2^E_g_(*ν*_1_)	1.71	18
(**3**)	[UO_2_(La-Maf)(H_2_O)_2_]·NO_3_	532(0.32)	Charge Transfer	__	112
(**4**)	[Ru(La-Maf)_2_(H_2_O)_2_]·H_2_O·Cl	652(0,34)	^2^T_2g_ → ^2^A_2g_(*ν*_1_)	1.73	103
(**5**)	[VO(La-Maf)_2_H_2_O]·H_2_O	733(0.47)	^2^B_1g_ → ^2^E_g_(*ν*_1_)	1.86	28
(**6**)	[UO_2_(La-Maf)_2_]·3H_2_O	538(0.37)	Charge Transfer	__	37
(**7**)	[Ru(Lb-Daf)(Cl)(H_2_O)]H_2_O	663(0.52)	^2^T_2g_ → ^2^A_2g_(*ν*_1_)	1.75	19
(**8**)	[VO(Lb-Daf)]·2H_2_O	736(0.29)	^2^B_1g_ → ^2^E_g_(*ν*_1_)	1.74	33
(**9**)	[UO_2_(Lb-Daf)]·2H_2_O	545(0.82)	ChargeTransfer	__	42
(**10**)	[Ru_2_(Lb-Daf)(Cl)_4_(H_2_O)_2_]·H_2_O	656(0.51)	^2^T_2g_ → ^2^A_2g_(*ν*_1_)	1.53	35
(**11**)	[(VO)_2_(Lb-Daf)(H_2_O)_2_]·2H_2_O	743(0.35)	^2^B_2_ → ^2^E(*ν*_1_)	__	18
(**12**)	[(UO_2_)_2_(Lb-Daf) (H_2_O)_4_]·2NO_3_	546(0.56)	Charge Transfer	__	136

a Absorption maxima in nm; molar absorptivities given in parentheses given in 10^4^ Lmol^-1^cm^-1^.

b Molar conductance (Ω^−1^ cm^2^ mol^−1^) was measured in 10^−3^ mol^−1^ DMF solvent.

The electronic spectral data for HLa-Maf and H_2_Lb-Daf ligands, show mainly three absorption bands at (267, 324 and 462 nm) for the former and (260, 315 and 453 nm) for the later. The first one is assigned to the transition of cyclopentadienyl rings in the two ligands [53], [54]. The second one, is due to π–π^*^, transition within the azomethin (C=N) group which is shifted to lower absorption on coordination, as a result of the coordination of the nitrogen atom of the azomethin, This shift can be attributed to overlap of the central-metal-d-orbital with the p-orbital of the donor atom. The later broad absorption band is assigned to charge transfer from iron to either the non-bonding or the antibonding orbitals of cyclopentadienyl rings. These absorption bands become weaker without remarkable shift on complex formation [33].

The electronic transition spectra of the ground state of Ru(III) is ^2^T_2g_, and the first excited doublet levels in the order of increasing energy are, ^2^A_2g_ and ^2^A_1g_ which arise from the t_2g_^4^eg^1^ configuration. In a d^5^ system, and especially in Ru(III) which is a relatively strong oxidizing agent, charge transfer bands are prominent in the low-energy region and obscure the weaker bands due to the d–d transition. The electronic spectra of Ru(III) complexes **1, 4, 7** and **10** exhibit mainly three bands at the ranges, (352–432 nm), (571–563 nm) and (643–663 nm), respectively. The first band is assigned to the d–d transition (^2^T_2g_ → ^2^A_2g_), while the second intense band is due to M–Lπ^*^ transition (^2^T_2g_ → ^2^E_g_). The last one is attributed to the inter-ligand transition or to MLCT bands (^2^T_2g_ → ^2^A_1g_). For the macrocyclic complex, [Ru_2_(Lb-Daf)(Cl)_4_(H_2_O)_2_]·H_2_O, **10**, the magnetic moment is lower than expected (1.53 B.M). This behavior can be explained by the presence of partial coupling interaction between the two electrons of the ruthenium ions in the complex [55]. The positions of the absorption bands as well as the magnetic susceptibility measurements indicate the presence of one unpaired electron and confirming a low-spin octahedral configuration Molar conductance indicate the neutral nature of complexes **1, 7** and **10** value at 36, 19 and 35 Ω^−1^ cm^2^ mole^−1^ respectively, while complex **4**, [Ru(La-Maf)_2_(H_2_O)_2_]·H_2_O·Cl, in agreement with the found electrolytic behavior (103 Ω^−1^ cm^2^ mole^−1^) [56].

The reflectance spectrum of each of the oxovanadium complexes, **2**, **8** and **11** shows well-defined two bands at 518 and 724 for the former, 512 and 736 nm for the second, 523 and 743 nm for latter assigned to ^1^B_2_ → ^2^E and ^1^B_2_ → ^2^A_1_ in a square-pyramid structure configuration with effective magnetic moments 1.71, 1.74 and 1.56 B.M. for complex, 2, 8 and 11 respectively [57], while for complex **5**, absorption at 733 and 547 nm can be assigned to ^2^B_2_ → ^2^E(*ν*_1_) and ^2^B_2_ → ^2^B_1_(*ν*_2_) corresponding to distorted octahedral geometry [58]. For the cyclic complex, [(VO)_2_(Lb-Daf)(H_2_O)_2_]. 2H_2_O **11**, its diamagnetic character offers a strong evidence for the presence of a V–V bond. This behavior can be explained by the presence of a strong metal–metal coupling interaction between the two parallel oxovandium ions in the complex. The unpaired electron in the 3d_xy_ of vanadium ion is overlapped with the d_xy_ orbital of the adjacent vanadium atom, which lead to direct spin–spin coupling [59]. The conductivity measurements indicate the non-electrolytic nature of complexes 2, **5, 8** and **11.** Complexes of (UO_2_)(VI) **3**, **6**, **9** and **11** are of diamagnetic nature, so there is no significant magnetic moment. The electronic spectra of the diamagnetic dioxouranium(VI) complex, show mainly two bands where, the absorption is tailing into the visible region, which produces the intense orange color. The first band is observed at the range 453–423 nm corresponding to charge transfer from equatorial donor atoms of the ligand to the uranyl ion. The second band is observed at the range 532–545 nm due to electronic transitions from apical oxygen atom to the f-orbitals of the uranyl atom characteristic of the uranyl moiety [58]. Molar conductance values in DMF were 112 and 136 Ω^−1^ cm^2^ mol^−1^, for **3**, [UO_2_(La-Maf)(H_2_O)_2_]·NO_3_ and **12**, [(UO_2_)_2_(Lb-Daf)(H_2_O)_4_]·2NO_3_ complexes, respectively, indicating the electrolytic nature for these compounds, on the other hand, for complex **6**, [UO_2_(La-Maf)_2_]·3H_2_O and **9**, [UO_2_(Lb-Daf)]·2H_2_O, the molar conductance value was 37 Ω^−1^ cm^2^ mol^−1^ for the former and 42 Ω^−1^ cm^2^ mol^−1^ for the later, indicating the non-electrolytic nature of theses complexes. With the aid of the elemental analysis and Infrared-spectra, the proposed configuration is expected to be a distorted octahedral for (UO_2_)(VI) complexes [59].

### ^1^H NMR spectra

^1^H NMR spectra of the free ligands HLa-Maf and H_2_Lb-Daf and their UO_2_(VI) complexes have been studied in DMSO-*d*_6_ without and with D_2_O and are characterized and summarized with their assignments in Table 4**.** The proton resonance of the thiol groups (3.51 ppm) for HLa-Maf ligands and the signals of the thiol groups for H_2_Lb-Daf (3.48 ppm) disappeared on adding D_2_O, which indicate that these protons are acidic. This observation has been further supported by the absence of resonances for SH protons in the spectra of [UO_2_(La-Maf)(H_2_O)_2_]·NO_3_, **3** and [UO_2_(Lb-Daf)]·2H_2_O, **9** complexes, indicates deprotonation of the —SH group of the Schiff bases and coordination of the sulfur atoms through the coordination to the metal atom [62]. Moreover, in the spectra of diamagnetic UO_2_(IV) complexes **4** and **10** the protons of methyl group bonded to the azomethine groups are shifted downfield compared to that of the free ligand as a result of chelation of azomethine group to metal ion, indicate that, the chelation of the ligand with the metal ions involves the nitrogen atom of the ligands. No appreciable change was observed in the chemical shift of the ferrocenyl or of the aromatic protons of the Schiff bases moieties on complexation. [63], [64].

**Table 4:** ^1^H NMR chemical shifts (*δ*, ppm) of the Schiff base, HLa-Maf and H_2_Lb-Maf, ligands and their UO_2_(IV) complexes **(3)** and **(9)**.

						
	Assignment		Chemical shift, *δ*_H_		Complexes	
			HLa-Maf	H_2_Lb-Daf	3, [UO_2_(LaMaf)(aH_2_O)_2_].NO_3_	9,[UO_2_(Lb-Daf)]2H_2_O
1	H(a)	[s,3H, CH_3_]	[2.23]	–	[2.53]	–
2	H(a′)	[s,6H, 2CH_3_]	–	[2.18]	–	[2.23]
3	H(b)	[s,1H-SH]	[3.52]	–	–	–
4	H(b′)	[s,2H-SH]	–	[3.48]	–	–
5	H(c, d)	[s,2H,Cp-H]	[4.74]	–	[4.75]	–
6	H(e, f)	[s,2H,Cp-H]	[4.33]	–	[4.31]	–
7	H(g, h, i, j, **k**)	[s,5H,Cp(C_5_H_5_)]	[4.06]	–	[4.08]	–
8	H(c′,g′)(d′,h′)′)(f′,j′)′)(d′,h′)	[m,8H,Cp2(C_5_H_4_)]	–	[4.82,4.78,4.62,4.45]	–	[4.82,4.79,4.62,4.43]
9	H(l, m, n, o)	[m,4H, Ar—H]	[6.77–7.48]	–	[6.63–7.77]	–
**10**	H(l′,m′,n′,o′)	[m,8H, Ar—H]	–	[6.67–7.42]	–	[6.61–7.46]

s = singlet; m = multiple, Cp = cyclopentiene ring.

a Signal due to coordinated water overlapped with Cp ring.

**Fig. 1:**
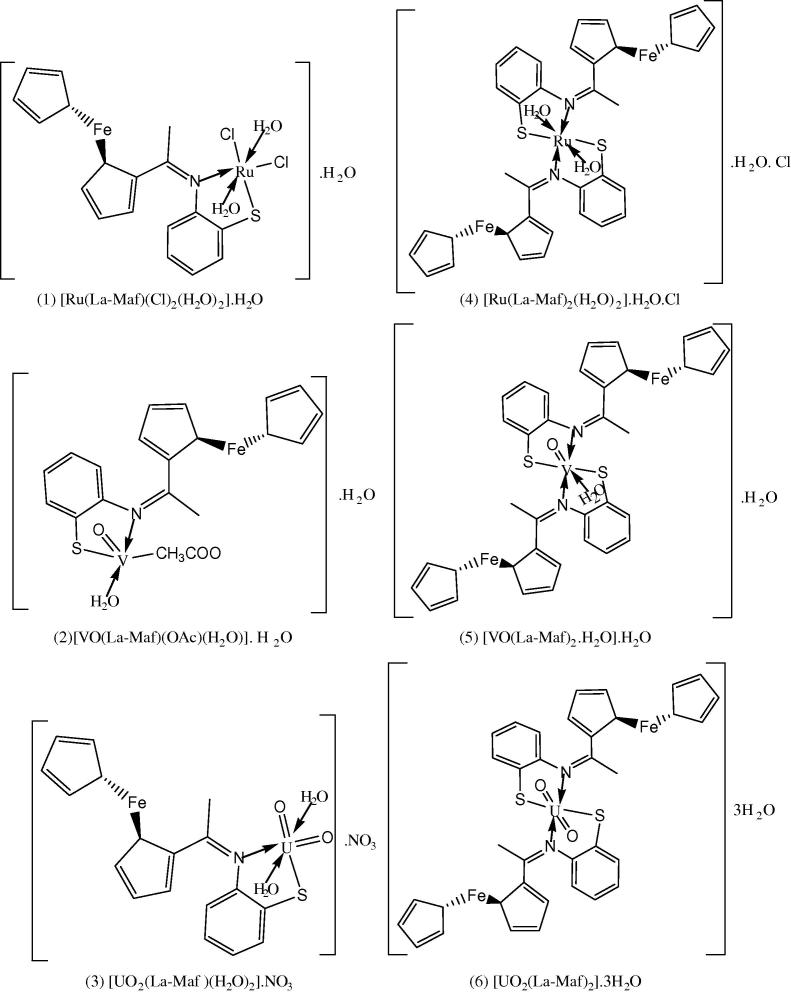
Representative structures of the metal complexes of the Schiff base, HLa-Maf.

**Fig. 2:**
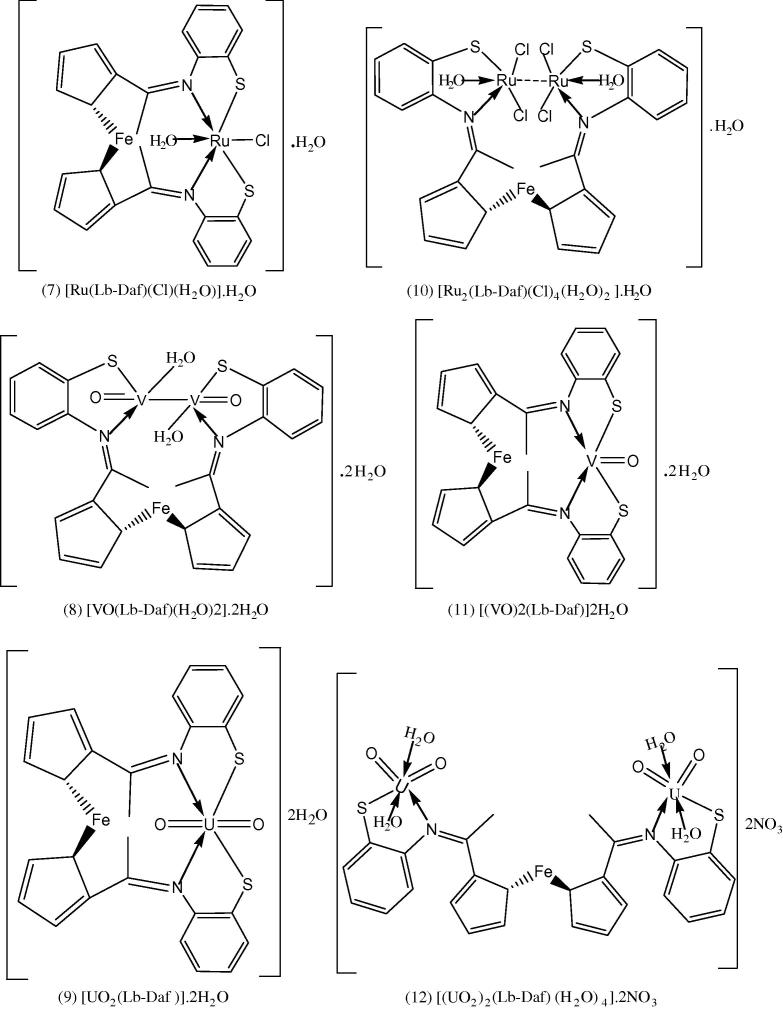
Representative structures of the metal complexes of the Schiff base, H_2_Lb-Daf.

Finally, from the interpretation of elemental analysis, spectral data and magnetic studies as well as the thermal analysis and molar conductivities measurements. It is possible to draw up the tentative structures of the transition metal complexes. Fig. 1, Fig. 2, depicts the proposed structures of the metal complexes.

#### Biological studies

The Schiff base, HLa-Maf and H_2_Lb-Daf and their metal complexes were evaluated for antimicrobial activity against one strain Gram-positive bacteria *S. aureus* and *P. fluorescens* as Gram-negative bacteria as well as one pathogenic fungus, as *F. oxysporum* The results of the biological studies of the ligand and the complexes are shown Table 5. The data are compared with standard antibiotics, *chloramphencol* as Gram-negative and *cephalothin* as standard reference for Gram-positive bacteria. Cycloheximide was used as antifungal standard reference. The in vitro antibacterial and antifungal activities demonstrated that complexes have higher antimicrobial activity in comparison with that of the ligand. According to Tweedy’s theory [65], chelation reduces the polarity of the metal atom because of partial sharing of its positive charge with a donor group and the possible π-electron delocalization over the whole chelate ring [66]. Such a chelation could enhance the lipophilic character of the central metal atom, which subsequently favors its permeation through the lipid layers of the cell membrane and blocking the metal binding sites on enzymes of microorganism [67], [68]. There are other factors which also increase the activity, such as solubility, conductivity and bond length between the metal and the ligand.

**Table 5:** Antimicrobial activity of HLa-Maf and H_2_Lb-Maf, ligands and their complexes.

Compound	Mean of zone diamete, mm mg mL^−1^^a^		
	Gram-positive bacteria^b^	Gram-negative bacteria^b^	Fungi^b^
	*Staphylococcus aureus*	*Pseudomonas phaseolicol*	*Fusarium oxysporium*
**I HLa**(**Maf**)	40 ± 0.4^c^	34 ± 0.2	36 ± 0.1
(1)[Ru(La-Maf)(Cl)_2_(H_2_O)_2_]·H_2_O	19 ± 0.1	21 ± 0.2	19 ± 0.2
(2)[VO(La-Maf)(OAc)(H_2_O)]·H_2_O	17 ± 0.2	20 ± 0.2	20 ± 0.2
(3)[UO_2_(La-Maf)(H_2_O)_2_]·NO_3_	16 ± 0.1	18 ± 0.3	18 ± 0.2
(4)[Ru(La-Maf)_2_(H_2_O)_2_]·H_2_O·Cl	38 ± 0.1	35 ± 0.3	36 ± 0.3
(5)[VO(La-Maf)_2_H_2_O]·H_2_O	32 ± 0.2	33 ± 0.1	36 ± 0.2
(6)[UO_2_(La-Maf)_2_]·3H_2_O	37 ± 0.2	32 ± 0.3	33 ± 0.2
**II H_2_Lb**(**Daf**)	31 ± 0.4^c^	30 ± 0.2	30 ± 0.1
(7)[Ru(Lb-Daf)(Cl)(H_2_O)] H_2_O	19 ± 0.2	17 ± 0.1	16 ± 0.3
(8)[VO(Lb-Daf)]·2H_2_O	20 ± 0.1	18 ± 0.1	17 ± 0.3
(9)[UO_2_(Lb-Daf)]·2H_2_O	17 ± 0.2	19 ± 0.1	21 ± 0.3
(10)[Ru_2_(Lb-Daf)(Cl)_4_(H_2_O)_2_]·H_2_O	26 ± 0.2	24 ± 0.1	27 ± 0.2
(11)[(VO)_2_(Lb-Daf)(H_2_O)_2_]·2H_2_O	23 ± 0.2	26 ± 0.3	23 ± 0.2
(12)[(UO_2_)_2_(Lb-Daf) (H_2_O)_4_]·2NO_3_	25 ± 0.2	24 ± 0.3	25 ± 0.2
**Antibiotic**^d^	**42**	**36**	**40**

a Calculated from three average values.

b Chloramphencol in the case of Gram-positive bacteria, Cephalothin in the case of Gram-negative bacteria and Cycloheximide in the case of fungi.

c Error limits, **±**.

d Control.

It is worth to mention that, ligands showed highly biological activity against the tested strains compared to the complexes. Structure activity relationships showed that complexes **4**,[Ru(La-Maf)_2_(H_2_O)_2_]·H_2_O·Cl, **5**[VO(La-Maf)_2_H_2_O]·H_2_O and **6** [UO_2_(La-Maf)_2_]·3H_2_O, of higher microbial activities. It may be due to the presence of two ferrocene ring, which might increase the lipophilic character of the molecules, which facilitate the crossing through the biological membrane of the microorganisms and thereby inhibit their growth. The result revealed also that the binuclear complexes enhances the antimicrobial activity rather than the mononuclear complexes [69], [70].

## Conclusion

The Schiff bases derived from the condensation between mono and diacetyl ferrocine and 2-aminobenzenthiol in different molar ratio, lead to series of mono and binuclear complexes of varying geometries. HLa-Maf acts as monobasic bidentate ligand while H_2_Lb-Daf acts as dibasic tetradentate chelate. From the infrared spectra, the chelation of the metal ions to the ligands occurs through the sulfur of the thiol and the nitrogen atoms of the azomethine groups of the ligands. The nitrate ions, acetate, chloride, crystalline or coordinated water molecules satisfy the other coordination sites to complete the geometry around the central metal ion. The spectral, magnetic studies and molar conductivity measurements of the metal complexes were used to determine the type of coordination and the geometry around the central metal ion. Acyclic and cyclic complexes exhibit either square pyramidal or octahedral. Synthesized Schiff base and their corresponding metal complexes were tested for the growth inhibitory activity against phytopathogenic bacteria and fungi, including some which are antibiotic resistant, makes it interesting for a practical use as antimicrobial agent. It is obvious that the activity become more pronounced when two feroccine rings are coupled and metal complexes are more toxic against bacteria and fungi in comparison to their parent compounds.
