# Supplementary material for: Synthesis, spectroscopic, coordination and biological activities of some organometallic complexes derived from thio-Schiff base ligands
Source: Spectrochim Acta A Mol Biomol Spectrosc. 2014 Jan 3;117:763–71. doi: 10.1016/j.saa.2013.06.078 (PMC3826106; doi:10.1016/j.saa.2013.06.078)
Supplement: Supplementary data 1 [file mmc1.docx]

**Synthesis, Spectroscopic, Coordination and Biological Activities of Some Organometallic Complexes Derived from Thio- Schiff Base Ligands**

WOLFGANG LINERT †AND AZZA A. ABOU-HUSSEIN‡

†Institute of Applied Synthetic Chemistry, Vienna University of Technology, Getreidemarkt, 9/163-AC, 1060 Vienna, Austria.

‡Faculty of Women for Arts, Science and Education, Ain Shams University, Heliopolis, Cairo, Egypt.

**Supplementary materials**

Index:

.
